# Spirituality, Religiosity and End-of-Life Decision-Making in the ICU: A Systematic Review

**DOI:** 10.1192/j.eurpsy.2026.10784

**Published:** 2026-07-31

**Authors:** D. Apostolakis, S. Georgakis, F. Tatsis, F. Veroniki, K. Stamatis, G. Papathanakos, N. Angelopoulos, M. Gouva, V. Koulouras

**Affiliations:** 1 Medicine, University of Ioannina, Ioannina; 2 Medicine, University of Thessaly, Larisa; 3 Scientific Laboratory of Psychology & Person-Centered Care, University of Ioannina, Ioannina, Greece

## Abstract

**Introduction:**

Spirituality and religiosity are increasingly acknowledged as significant determinants influencing attitudes and clinical practices surrounding end-of-life (EoL) decision-making in intensive care units (ICUs). However, the existing body of evidence remains fragmented, frequently confined to particular stakeholder groups or situated within narrowly defined cultural and regional contexts.

**Objectives:**

This study aimed to systematically synthesize evidence on the influence of spirituality and religiosity on stakeholders’ attitudes and clinical practices in the ethically complex domain of EoL decision-making in ICUs.

**Methods:**

A comprehensive PubMed search was undertaken in August 2025, in accordance with PRISMA 2020 guidelines. Eligible studies comprised observational designs and qualitative research addressing adult stakeholders in whom spirituality or religiosity was examined as a factor influencing EoL decision-making. Studies conducted in pediatric populations, case reports, and non-empirical contributions were excluded. Owing to the heterogeneity of study designs and outcomes, the findings were synthesized narratively.

**Results:**

Of 246 records screened, eight studies originating from Europe, Asia, and the USA met the inclusion criteria. Survey data suggested that physicians with stronger religious affiliations were more reluctant to withdraw life-sustaining treatment and more likely to regard such actions as ethically problematic. Qualitative investigations demonstrated that surrogates’ spirituality influenced acceptance of comfort-focused care. Furthermore, strong support from religious communities and clergy was associated with more aggressive interventions and higher ICU mortality, whereas spiritual support provided by medical teams was correlated with greater hospice referrals, reduced use of aggressive treatment, and fewer ICU deaths.

**Conclusions:**

Spirituality and religiosity shape the full spectrum of end-of-life (EoL) decision-making in heterogeneous and context-dependent ways. Higher levels of religiosity were associated with preferences for more aggressive interventions, whereas spiritual support provided by medical teams was associated with more patient-centered outcomes. Future research should explore these dynamics across diverse cultural settings to support ethically consistent and clinically responsive approached to EoL decision-making.

**Disclosure of Interest:**

None Declared

